# The Dynamic Changes in Maternal Thyroid Parameters Across the Three Trimesters and Their Differential Effects on the Occurrence of Adverse Obstetric Outcomes

**DOI:** 10.2174/0118715303351648250120113846

**Published:** 2025-03-04

**Authors:** Zheng Yang, Jingli Sun, Jinguang Wang, Xiaohui Jin, Yiyang Gao, Zhenyu Lin, Chenling Fan, Dongdong Luo, Deping Wang, Weiping Teng, Zhongyan Shan, Jing Li

**Affiliations:** 1 Department of Endocrinology and Metabolism, Institute of Endocrinology, Liaoning Provincial Key Laboratory of Endocrine Diseases, NHC Key Laboratory of Diagnosis and Treatment of Thyroid Diseases, The First Hospital of China Medical University, Shenyang, 110001, P. R. China;; 2 Department of Endocrinology, Nanyang Central Hospital, Henan 473000, P. R. China;; 3 Department of Obstetrics and Gynecology, General Hospital of Northern Theater Command (Heping Campus), Shenyang, 110001, P. R. China

**Keywords:** Anti-thyroid peroxidase antibody, anti-thyroglobulin antibody, thyroid dysfunction, obstetric outcomes, pregnancy, trimester

## Abstract

**Objective:**

Thyroid parameters undergo significant dynamic changes during pregnancy. This study aimed to comprehensively analyze the impact of abnormal thyroid parameters in each trimester on the incidence of common adverse obstetric outcomes.

**Methods:**

Blood samples drawn for thyroid parameters in each trimester during the antenatal period were determined after the participants gave birth. Serum thyrotropin, free thyroxine, free triiodothyronine, anti-thyroid peroxidase antibody (TPOAb), and anti-thyroglobulin antibody (TgAb) levels were tested using electrochemiluminescence immunoassays.

**Results:**

Among all the participants, TAI and hypothyroxinemia in the first trimester (T1) were significantly related to an increased risk of gestational hypertension (OR=5.136, 95% CI 1.537-17.158 and OR=7.683, 95% CI: 1.890-31.229, respectively). Additionally, subclinical hypothyroidism in T1 was independently associated with a higher risk of postpartum hemorrhage (OR = 38.063, 95% CI 2.091-692.834). Besides, subclinical thyrotoxicosis in T1 showed a significant correlation with a raised risk of small for gestational age (OR=14.650, 95% CI 1.221-175.760). Among euthyroid women during the whole pregnancy, either TPOAb+ or TgAb+ in the third trimester was an independent risk factor of premature birth (OR=5.092, 95% CI 1.059-24.481) and low birth weight (OR=8.165, 95% CI 1.717-38.824), respectively.

**Conclusion:**

Our findings indicate the importance of screening thyroid parameters in early pregnancy and the need to dynamically monitor these parameters throughout the entire pregnancy.

## 1 INTRODUCTION

In pregnancy, a series of physiological alterations in maternal Thyroid Hormone (TH) levels occur to meet the metabolic requirements and energy homeostasis for the mother and fetus [1]. Thyroid Dysfunctions (TDs) commonly affect women of childbearing age [2]. The influences of subclinical TDs on adverse pregnancy-related complications and fetal outcomes are issues generally focused on but lackconsistent conclusions. Moreover, women with positive anti-thyroid peroxidase antibody (TPOAb) and/or anti-thyroglobulin antibody (TgAb) are susceptible to developing TDs during pregnancy and suffering from adverse obstetrical complications [2]. It is well known that the serum levels of Free Triiodothyronine (FT3), Free Thyroxine (FT4), Thyroid Stimulating Hormone (TSH), TPOAb, and TgAb undergo changes throughout the entire gestational days [3-5]. It has been reported that TDs and Thyroid Autoimmunity (TAI) are potentially associated with a few poor maternal and fetal outcomes, including Gestational Hypertension (GHT), Gestational Diabetes Mellitus (GDM), miscarriage, Premature Birth (PTB) and postpartum thyroiditis [6-10]. However, no study has consecutively observed the prevalence rates of TDs during the whole pregnancy. Furthermore, most previous studies investigating thyroid disorders (TDs) or TAI in relation to adverse pregnancy or obstetric outcomes have focused on only one or two specific periods during pregnancy, or have not evaluated TDs across all three trimesters [2, 5, 11-15]. Thus, the above two issues have been the main purposes of this retrospective observational study based on a maternal-fetal cohort established in Northeast China. The findings have been of much value in guiding the managements of TDs/TAI during pregnancy.

## 2 MATERIAL AND METHODS

### 2.1 Study Design and Participants

**Fig. (1):**
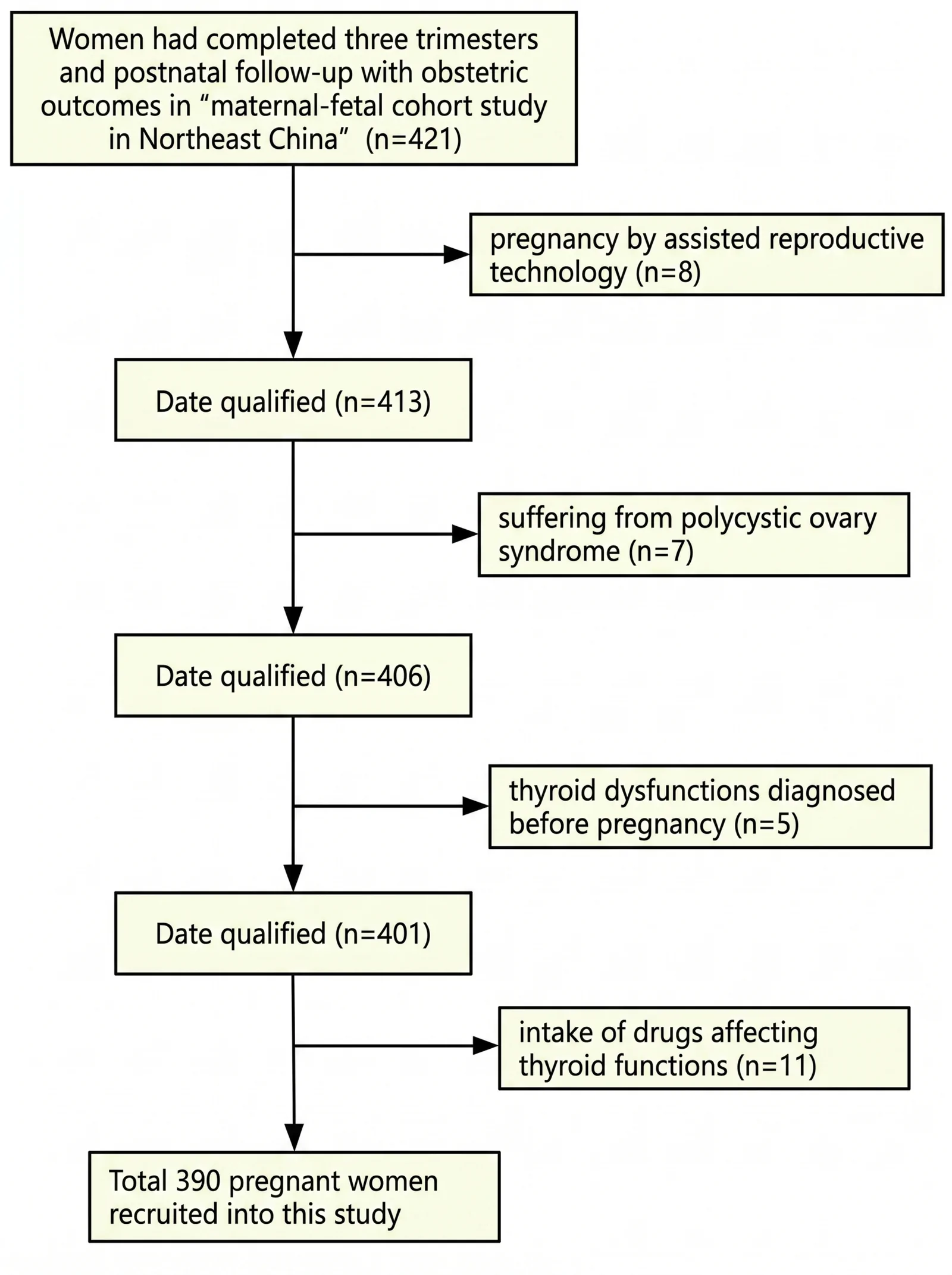
Flowchart of participant recruitment into this study.

All the participants in this study were recruited from those who were enrolled in the “maternal-fetal cohort study in Northeast China (MFCNC)” at the 6^th^~14^th^ week of gestation and had completed three trimesters and postnatal follow-up with obstetric outcomes in the Department of Obstetrics and Gynecology, General Hospital of Northern Theater Command (Heping Campus). The latter was one of the 4 centers where the MFCNC study had been conducted from January 2018 to June 2022. The inclusion criteria for this study were as follows: 1) Chinese women who had resided in Liaoning province, an iodine-sufficient area, for more than 6 months, 2) 20-40 years old, 3) with a singleton pregnancy, 4) follow-up information and outcomes of pregnancy available, and 5) serum FT3, FT4, TSH, TPOAb and TgAb levels all available in the whole three trimesters of pregnancy (first trimester of pregnancy, < 14^th^ week, second trimester of pregnancy, ≥ 14^th^ and < 28^th^ week, and third trimester of pregnancy, ≥ 28^th^ week) [16-18]. The exclusion criteria included pregnancies by assisted reproductive technologies, pregnant women with polycystic ovary syndrome, pre-existing thyroid diseases (excluding TAI), and intake of drugs affecting thyroid functions. According to the above criteria, a total of 390 pregnant women were finally included in this study (Fig. **1**). An Oral Glucose Tolerance Test (OGTT) was performed at 24-28 weeks of gestation. All subjects understood and signed the written informed consent form before participating.

### 2.2 Follow-up and Data Collection

All the participants were followed up during each trimester. At each visit, all subjects were given a questionnaire about general information, such as gestational age, smoking, vitamin and food intake, disease suffering, and drug usage. Besides, their fasting blood samples were collected, and all the sera were stored at -80°C for later detection of thyroid functions and auto-antibodies after birth. Among the 390 participants, 338 had given birth in the General Hospital of Northern Theater Command (Heping Campus). The information about maternal and neonatal outcomes was obtained by inquiring about the medical records of the inpatient system. The remaining 52 participants who had delivered in other hospitals had a telephonic follow-up to obtain final obstetric outcomes at 8-12 weeks postpartum.

### 2.3 Serum Sample Collection and Tests

Maternal serum samples were collected during each trimester. After all pregnant women gave birth, Fasting Blood Glucose (FBG) was tested using the hexokinase method with Roche COBAS8000 C702 automatic biochemical immunoassay analyzer (Roche Diagnosis, Switzerland), serum FT4, FT3, TSH, TPOAb, and TgAb were measured using electrochemiluminescence immunoassays with Cobas Elecsys 601 (Roche Diagnostics, Switzerland). The intra-assay Coefficients of Variation (CV) of serum FT4, FT3, TSH, TPOAb, and TgAb were 1.4%-2.9%, 1.5-2.2%, 1.5%-8.6%, 2.2%-6.3%, and 2.4%-5.6%, respectively. The inter-assay CV values were 2.7%-6.6%, 1.7-2.8%, 1.8%-8.7%, 5.2%-8.2%, and 2.1%-6.9%, respectively [19]. The diagnosis of TAI was based on serum levels of TPOAb (> 34 IU/ml) and/or TgAb (> 115 IU/ml) in T1 [19]. Hypothyroxinemia was diagnosed when serum FT4 level was found below the 2.5^th^ percentile while serum TSH was between the 2.5^th^ and 97.5^th^ percentiles. Hypothyroidism was diagnosed if serum FT4 level was below the 2.5^th^ percentile and TSH concentration above the 97.5^th^ percentile, which was trimester-specific. In the patients with Subclinical Hypothyroidism (SCH), serum FT4 level was between the 2.5^th^ and 97.5^th^ percentiles, while serum TSH was higher than the 97.5^th^ percentile. Thyrotoxicosis was defined as serum FT4 level above the 97.5^th^ percentile and serum TSH below the 2.5^th^ percentile. Subclinical Thyrotoxicosis (SCT) was indicated when serum FT4 level was between the 2.5^th^ and 97.5^th^ percentiles while serum TSH level was below the 2.5^th^ percentile. Trimester-specific reference ranges of serum FT4 and TSH in pregnant women were listed in Supplementary Table **1**, including the 2.5^th^, 50^th,^ and 97.5^th^ percentiles [20].

### 2.4 Definitions of Adverse Maternal and Neonatal Outcomes

The adverse maternal and neonatal outcomes were defined as follows. GHT was defined as persistent Blood Pressure (BP) of 140/90 mmHg or more after 20 weeks gestation (at least two BP readings) in previously normotensive women [21]. GDM was referred to FBG level ≥ 5.1 mmol/l, 1h Post-load Blood Glucose (PBG) ≥ 10.0 mmol/l and/or 2h PBG ≥ 8.5 mmol/l [22]. Oligohydramnios was diagnosed when the amount of amniotic fluid was < 400 ml. Premature Rupture of Membranes (PROM) was referred to as the rupture of membranes before the onset of labor. Postpartum hemorrhage was defined as more than 500 ml of blood loss within the first 24h after vaginal childbirth. PTB was diagnosed when delivery occurred before 37 weeks of gestation while after 28 weeks of gestation. The infants with Low Birth Weight (LBW) presented birth weight < 2.5 kg, while those with macrosomia ≥ 4 kg. Fetal distress was indicated if there was a lack of oxygen supply based on some symptoms: fetal heart rate of > 160 or < 120 beats/min, abnormal fetal movement, presence of meconium, fetal abnormalities or complications, and fetal scalp pH of 7.2 [23, 24]. Small for Gestational Age (SGA) is defined as the lowest 10^th^ population-specific percentile of birth weight standardized to gestational age and sex [25].

### 2.5 Statistical Analyses

All statistical analyses were performed utilizing SPSS software (version 24.0, IBM Corporation, NY, USA). Independent sample t test was used for comparison between only the two groups when normally distributed continuous variables were analyzed. Mann-Whitney U-test was used for comparison between only the two groups when continuous variables did not conform to the normal distribution. Analysis of variance was adopted for comparisons among groups when normally distributed continuous variables were analyzed. The Kruskal-Wallis test was used for comparisons among groups when continuous variables did not conform to the normal distribution, and the Bonferroni test was used for post-hoc analysis. The chi-squared test or Fisher’s exact test was used for comparisons of categorical variables. Binary logistic regression with a stepwise procedure was used to assess the association between thyroid diseases and a series of adverse maternal and fetal outcomes. The trend test was used to examine the titer-dependent associations between serum TPOAb, TgAb levels, and the prevalence of PTB and LBW in euthyroid women. *P* < 0.05 was considered statistically significant.

## 3 RESULTS

### 3.1 Baseline Characteristics, Thyroid Functions, and Obstetric Outcomes of All the Subjects

Three hundred and ninety gravidae were recruited into this study, including 46 (11.8%) TAI women (TPOAb^+^ and/or TgAb^+^) and 344 (88.2%) non-TAI ones (TPOAb^-^TgAb^-^). There was no significant difference in the baseline characteristics between TAI and non-TAI women except for the proportion of those with an alcohol drinking history (Table **1**). TAI women showed markedly more susceptibility to developing GHT and delivering LBW newborns. In addition, there were gradually decreasing trends in serum FT4 and FT3 levels with an upward tendency in serum TSH levels from the T1 to T3 in all the participants (Table **1**). In TAI pregnant women, the two thyroid auto-antibodies in the serum were also significantly decreased in T3 when compared with those in T1 (Table **1**).

### 3.2 The Changes in the Prevalence Rates of TDs During Three Trimesters of Pregnancy

**Fig. (2):**
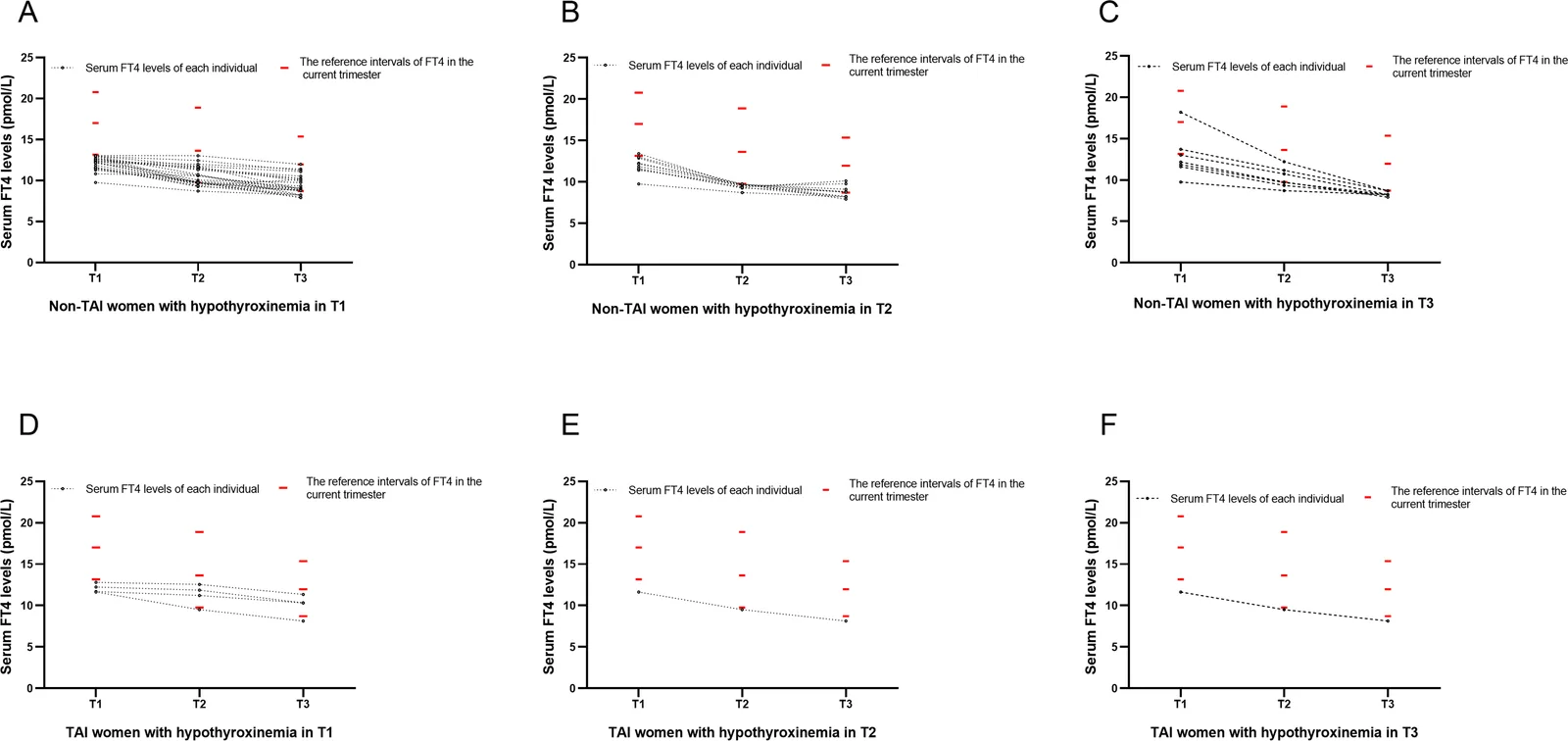
**(A)** Changes in FT4 levels throughout pregnancy in non-TAI pregnant women with hypothyroxinemia in T1. (**B**) Changes in FT4 levels throughout pregnancy in non-TAI pregnant women with hypothyroxinemia in T1. (**C**) Changes in FT4 levels throughout pregnancy in non-TAI pregnant women with hypothyroxinemia in T1. (**D**) Changes in FT4 levels throughout pregnancy in non-TAI pregnant women with hypothyroxinemia in T1. (**E**). Changes in FT4 levels throughout pregnancy in TAI pregnant women with hypothyroxinemia in T2. (**F**) Changes in FT4 levels throughout pregnancy in TAI pregnant women with hypothyroxinemia in T3. All the subjects were divided into non-TAI (TPOAb^-^TgAb^-^) and TAI (either TPOAb^+^ >34IU/ml or TgAb^+^ >115 IU/ml) groups. Thyroid functions and auto-antibodies were determined using electrochemiluminescence immunoassay (Roche). The reference intervals were expressed as the 2.5^th^, 50^th^, and 97.5^th^ percentiles. All the abbreviations as described in Tables **1** and **2**.

**Fig. (3):**
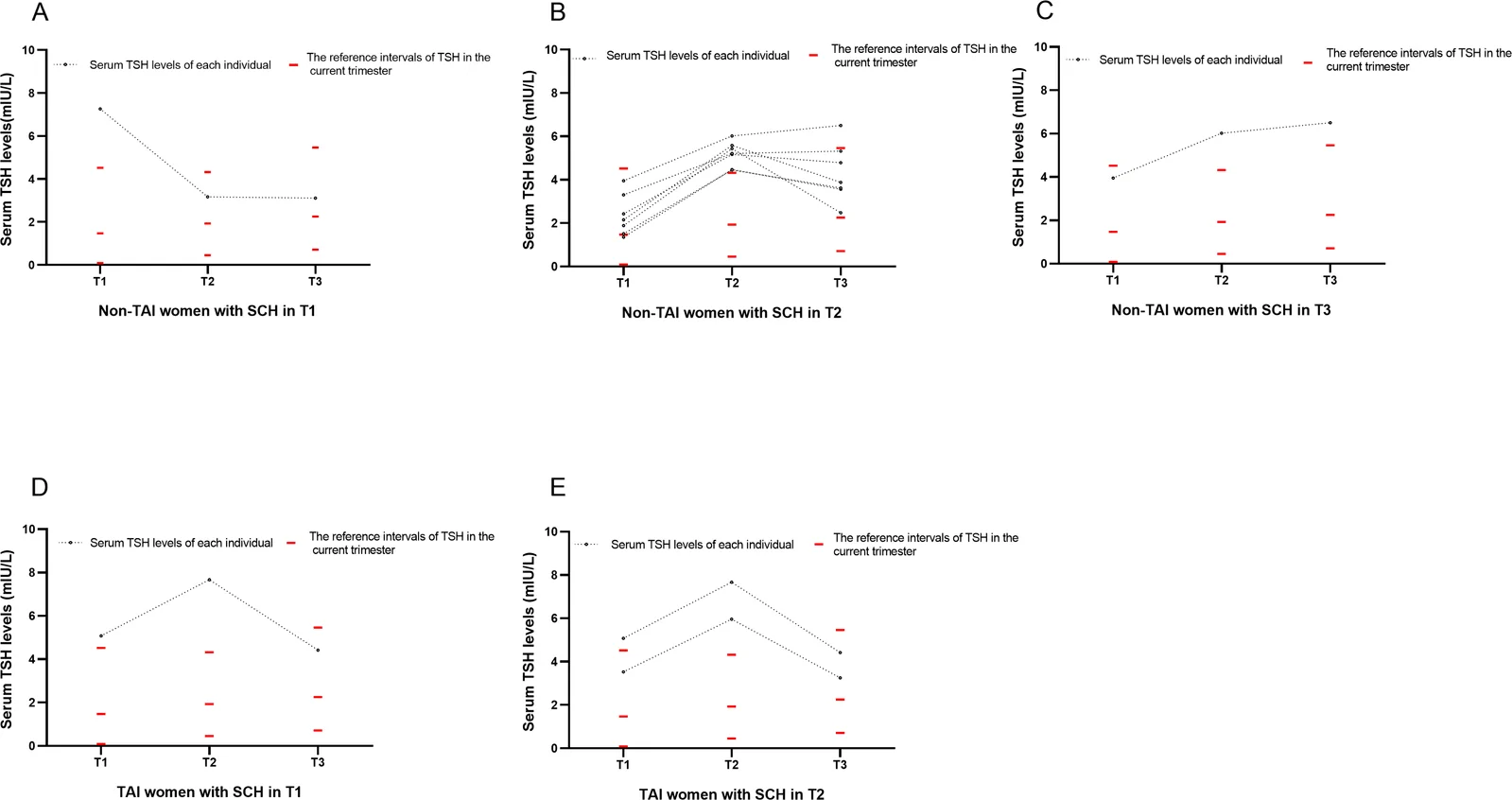
**(A)** Changes in FT4 levels throughout pregnancy in TAI pregnant women with hypothyroxinemia in T3. (**B**) Changes in TSH levels throughout pregnancy in non-TAI pregnant women with SCH in T2. (**C**) Changes in TSH levels throughout pregnancy in non-TAI pregnant women with SCH in T2. (**D**) Changes in TSH levels throughout pregnancy in TAI pregnant women with SCH in T1. (**E**) Changes in TSH levels throughout pregnancy in TAI pregnant women with SCH in T2. All subjects were divided into non-TAI and TAI groups as described in Fig. (**2**). The reference intervals were expressed as the 2.5^th^, 50^th^, and 97.5^th^ percentiles. None of the TAI women in T3 had SCH. All the abbreviations as described in Table **1**.

**Fig. (4):**
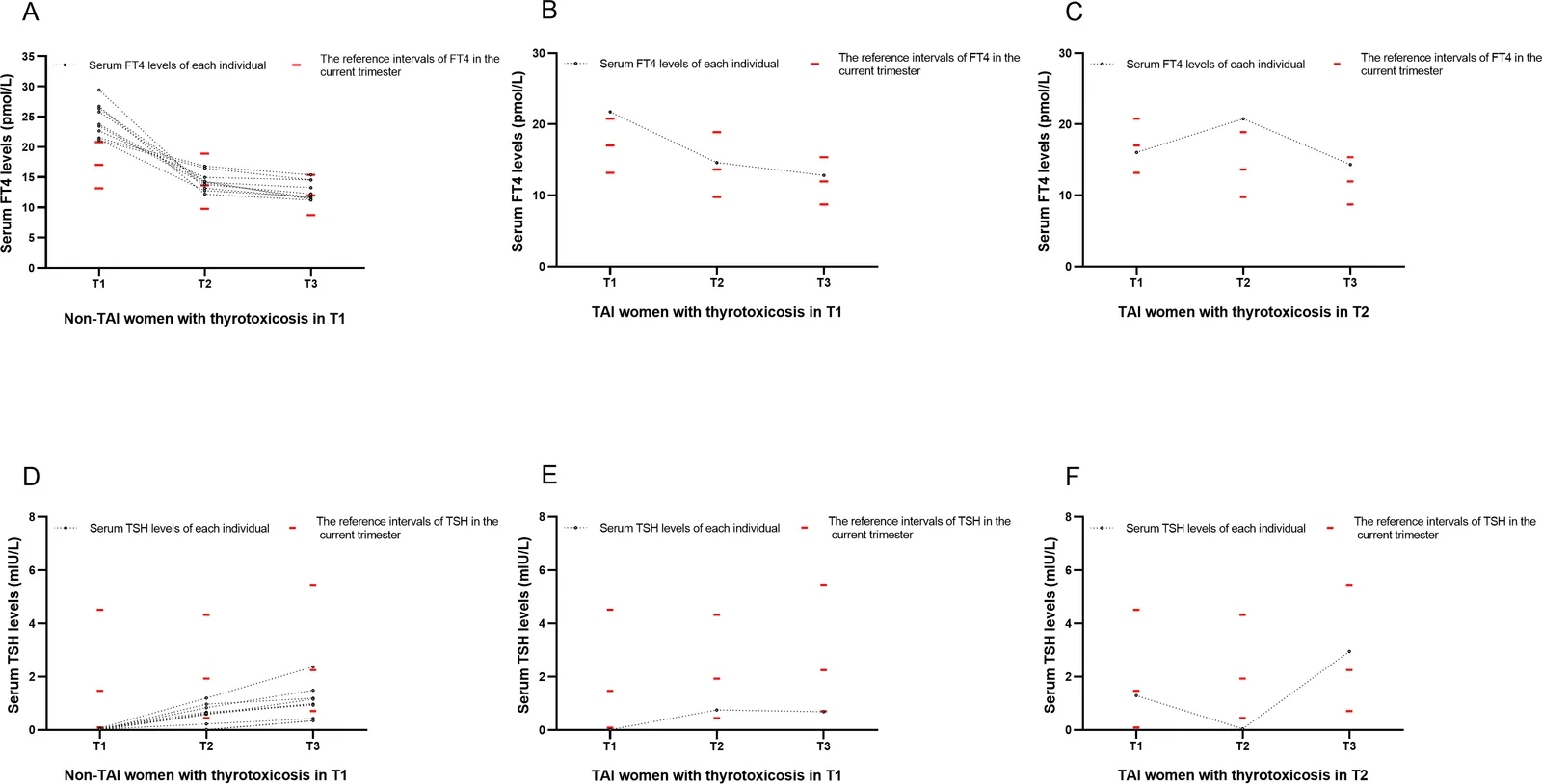
**(A)** Changes in FT4 levels throughout pregnancy in non-TAI pregnant women with thyrotoxicosis in T1. (**B**) Changes in FT4 levels throughout pregnancy in TAI pregnant women with thyrotoxicosis in T1. (**C**) Changes in FT4 levels throughout pregnancy in TAI pregnant women with thyrotoxicosis in T1. (**D**) Changes in FT4 levels throughout pregnancy in TAI pregnant women with thyrotoxicosis in T1. (**E**) Changes in FT4 levels throughout pregnancy in TAI pregnant women with thyrotoxicosis in T1. (**F**) Changes in TSH levels throughout pregnancy in TAI pregnant women with thyrotoxicosis in T2. All subjects were divided into non-TAI and TAI groups as described in Fig. (**2**). The reference intervals were expressed as the 2.5^th^, 50^th^, and 97.5^th^ percentiles. All the abbreviations as described in Table **1**.

**Fig. (5):**
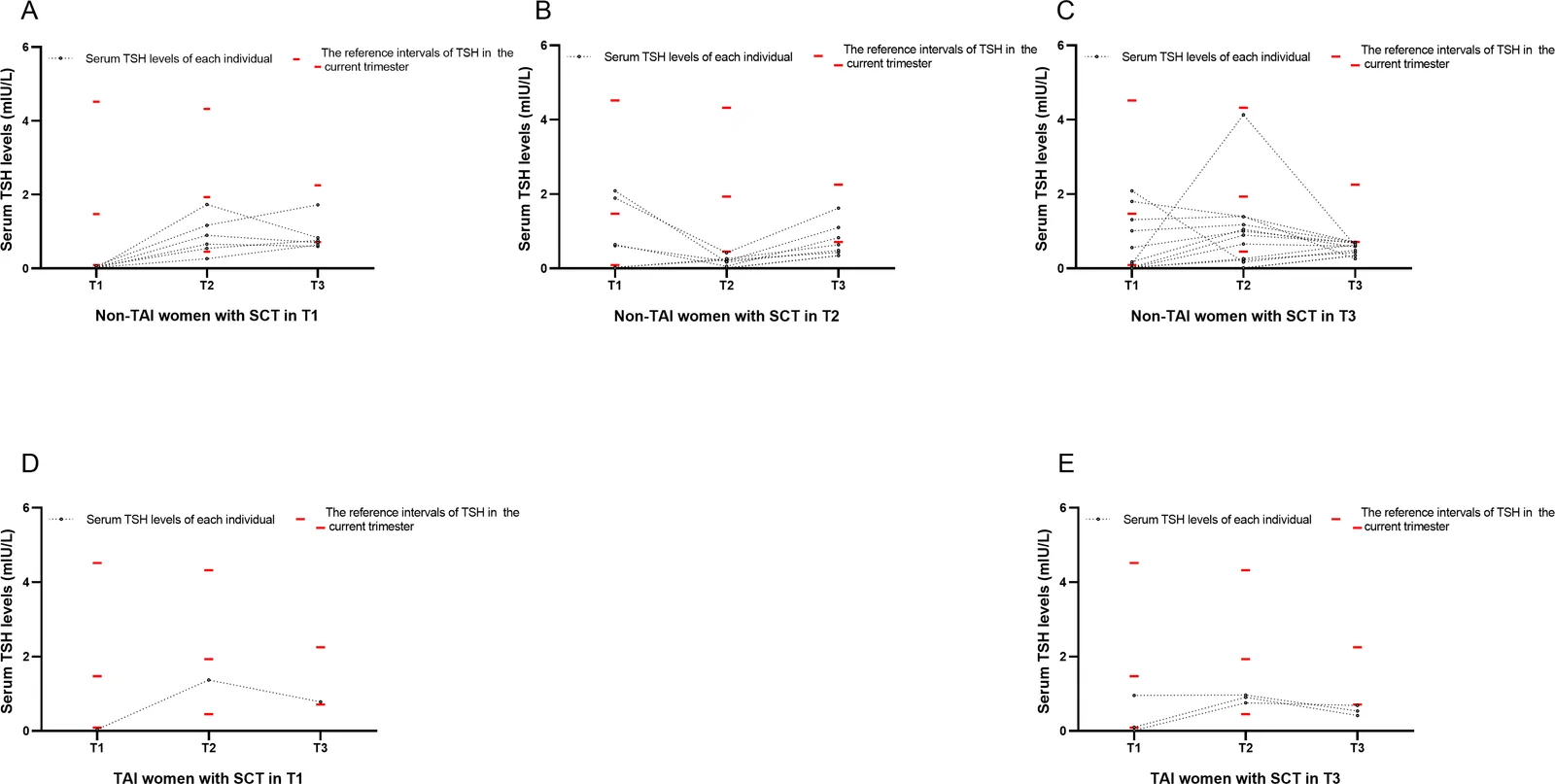
**(A)** Changes in TSH levels throughout pregnancy in non-TAI pregnant women with SCT in T1. (**B**) Changes in TSH levels throughout pregnancy in non-TAI pregnant women with SCT in T2. (**C**) Changes in TSH levels throughout pregnancy in non-TAI pregnant women with SCT in T3. (**D**) Changes in TSH levels throughout pregnancy in TAI pregnant women with SCT in T1. (**E**) Changes in TSH levels throughout pregnancy in TAI pregnant women with SCT in T1. All subjects were divided into non-TAI and TAI groups as described in Fig. (**2**). The reference intervals were expressed as the 2.5^th^, 50^th^, and 97.5^th^ percentiles. None of the TAI women in T2 had SCT. All the abbreviations as described in Tables **1** and **2**.

Thyroid function was dynamically monitored throughout pregnancy. Based on the trimester-specific reference ranges, most of them remained euthyroid during the whole pregnancy, whether or not expressing thyroid auto-antibodies. However, the percentage of euthyroidism exhibited a significant increasing trend while that of thyrotoxicosis gradually decreased from T1 to T3 among non-TAI women but not in TAI ones (Table **2**). Hypothyroxinemia was the most common thyroid disease among non-TAI women in T1 and T2, whereas the prevalence of SCT was highest among both TAI and non-TAI women in T3 (Table **2**). Approximately 1/2 of women with hypothyroxinemia in T1 were euthyroid in T2, while 2/3 recovered to euthyroidism in T3 (Fig. **2**). There was only one woman with hypothyroidism. She had TAI and exhibited euthyroidism in T2 and T3. Among the two females with SCH in T1, the one with TAI still had SCH in T2, while other non-TAI women recovered to euthyroidism in T2 (Fig. **3**). Among all the women with thyrotoxicosis in T1, 27.3% had SCT, 72.7% recovered to euthyroidism in T2, while 63.6% completely recovered in T3 (Fig. **4**). Among those women with SCT in T1, 85.7% recovered to euthyroidism in T2, while 57.1% recovered to euthyroidism in T3 (Fig. **5**). None of the participants had hypothyroidism during T2 or T3. Additionally, neither the participants nor the non-TAI women had thyrotoxicosis during T2 or T3, and none of the TAI women had subclinical hypothyroidism (SCH) during T3 or Subclinical Thyrotoxicosis (SCT) during T2.

### 3.3 The Relationships Between Maternal TDs During Each Trimester and Adverse Obstetric Outcomes Among All the Participants

Among non-TAI females, the prevalence rate of oligohydramnios was significantly increased in those with hypothyroxinemia in T1, and that of postpartum hemorrhage was significantly raised in those with SCH in T1 (Table **3**). Among TAI ones, the prevalence rate of GHT was significantly raised in those with hypothyroxinemia in T1, and that of oligohydramnios was significantly raised in those with SCH and SCT in T1 (Table **3**). Furthermore, binary logistic regression with a stepwise procedure was used to analyze the relationships between maternal TDs during each trimester and adverse obstetric outcomes. Maternal age at enrollment, maternal Body Mass Index (BMI) before pregnancy, parity, nationality, history of smoking, alcohol/tea drinking, and coffee use were included as confounding factors. Among all the participants, hypothyroxinemia in T1 and TAI were significantly associated with an increased risk of GHT (OR 7.683, 95% CI 1.890-31.229 and OR 5.136, 95% CI 1.537-17.158, respectively). SCH in T1 was independently associated with a higher risk of postpartum hemorrhage (OR 38.063, 95% CI 2.091-692.834), while SCT in T1 showed a significant correlation with a raised risk of SGA (OR 14.650, 95% CI 1.221-175.760). It was found that every 1.5 pmol/L decrease in serum FT4 level below the normal lower limit (13.15 pmol/L) in T1 was related to a 1.8-fold higher risk of GHT (*P* = 0.023), and every 1.5 mIU/L increase in serum TSH level above the normal upper limit (4.52 mIU/L) in T1 was associated with a 2.1-fold higher risk of postpartum hemorrhage (*P* = 0.022).

### 3.4 The Relationships between Expressions of TPOAb and TgAb During Each Trimester and Adverse Obstetric Outcomes Among Euthyroid Women

**Fig. (6):**
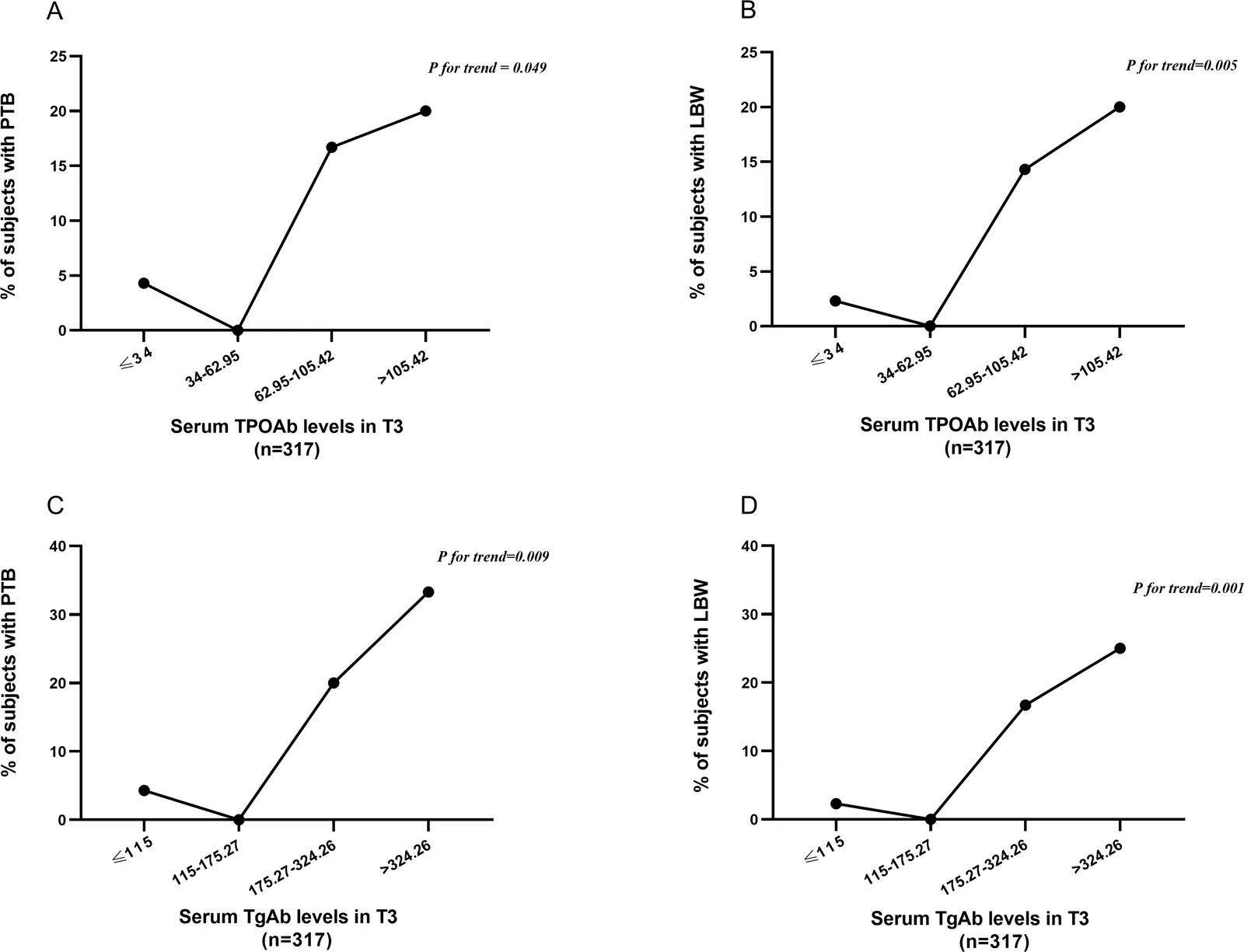
**(A)** The titer-dependent relationship between serum expressions of TPOAb in T3 and the prevalence rates of PTB in euthyroid women. (**B**) The titer-dependent relationship between serum expressions of TPOAb in T3 and the prevalence rates of LBW in euthyroid women. (**C**) The titer-dependent relationship between serum expressions of TgAb in T3 and the prevalence rates of PTB in euthyroid women. (**D**) The titer-dependent relationship between serum expressions of TgAb in T3 and the prevalence rates of PTB in euthyroid women. Four grades were divided based on the normal reference ranges (< 34 IU/ml for TPOAb, < 115 IU/ml for TgAb) and the tertiles of serum TPOAb and TgAb levels above the upper limit of reference range in those euthyroid pregnant women. All the abbreviations as described in Table **1**.

The potential adverse effects of TPOAb and TgAb on obstetric outcomes were further analyzed in euthyroid women in order to avoid the interactions between thyroid auto-antibody production and TDs. Maternal age at enrollment, maternal BMI before pregnancy, parity, nationality, history of smoking, alcohol/tea drinking, and coffee use were included as confounding factors. Binary logistic regression with stepwise procedure showed that the pregnant women with either TPOAb^+^ or TgAb^+^ (TPOAb^+^/TgAb^+^, *i.e.,* TAI) in T3 had an increased risk of PTB (OR 5.092, 95% CI 1.059-24.481) and LBW (OR 8.165, 95% CI 1.717-38.824), respectively. However, either serum TPOAb^+^TgAb^-^, TPOAb^-^TgAb^+,^ or TPOAb^+^TgAb^+^ in each trimester was not found to be significantly related to the occurrence of GHT, GDM, oligohydramnios, PROM, postpartum hemorrhage, macrosomia, fetal distress, or SGA. Based on the upper normal limits, both serum TPOAb and TgAb expressions were divided into four grades for titer-dependent trend analyses (Fig. **6**). With the increase of serum TPOAb titer > 34 IU/ml and serum TgAb titer > 115 IU/ml, the prevalence rates of PTB and LBW in the offspring of euthyroid females were progressively increased (Fig. **6**).

## 4 DISCUSSION

The longitudinal changes in thyroid parameters of women across the whole gestation were characterized in this study. It was observed that FT4 and FT3 levels decreased gradually with gestation and that the level of TSH increased in T2 and T3 compared to that in T1, which is consistent with previous studies [14, 26]. It suggests that TSH may reach a plateau in T2. Serum titers of TPOAb and TgAb were found with a decreasing trend throughout the pregnancy in TAI females in this study, which was consistent with the prevenient findings [4].

In this natural population-based cohort study, the proportion of TAI women was 11.8%, in agreement with the previous findings [2, 27]. Hypothyroxinemia was the most common thyroid disease among non-TAI women in T1 and T2, whereas the prevalence of SCT was highest among both TAI and non-TAI women in T3. The relationships between maternal TDs during each trimester and adverse obstetric outcomes were further analyzed in this natural population-based cohort study. Binary logistic regression analysis showed that TAI and hypothyroxinemia in T1 were risk factors for GHT. Lai *et al*. found that women with hypothyroidism during early pregnancy had an increased risk of GHT [6]. Su *et al*. found that hypothyroxinemia in T2 was a risk factor for GHT [28]. Another research showed a negative relationship between preeclampsia and GHT occurrence and maternal FT4 level in T3 [29]. In addition, they found that the levels of adenosine monophosphate, 5’-deoxyadenosine, and adenosine obviously declined in pregnant women with lower maternal FT4 levels [29]. Since adenosine is a vasodilator hormone in cerebral, muscular, and coronary systems, the decreased FT4 level in hypothyroid women may contribute to the occurrence of GHT by downregulating serum adenosine. In addition, SCH in T1 was found as an independent risk factor for postpartum hemorrhage in this study. Kiran *et al*. reported that serum TSH level > 2.5 mIU/L before conception and in T3 was significantly associated with postpartum hemorrhage. However, they did not find a correlation between serum TSH levels in T1 and postpartum hemorrhage [15]. Feldthusen *et al*. also showed that postpartum hemorrhage was more frequent in the SCH group than in the euthyroid group in T3 [30]. Thus, further studies are needed to explore the mechanisms by which thyroid parameters affect the occurrence of postpartum hemorrhage. Finally, SCT in T1 was shown as an independent risk factor of SGA in our study. This was not entirely in agreement with previous studies. Monen *et al*. showed that TSH ≥ 97.5th percentile was an increased risk of SGA [12], Vrijkotte *et al*. found that maternal SCH was related to an increased risk of LGA, while they did not find a relationship between maternal TSH level and SGA [31]. Therefore, the specific mechanisms need to be further studied. There was no correlation found between other TDs in the three trimesters and the adverse pregnancy outcomes (*e.g.*, GDM, PROM, and macrosomia) investigated in this study. These results suggest that more attention should be paid to hypothyroxinemia, SCH, and SCT in T1 among those females without chronic diseases or pre-existing thyroid or autoimmune disorders except TAI.

Besides, binary regression analysis of only euthyroid women showed that TPOAb^+^/TgAb^+^ in T3 was an independent risk factor for both PTB and LBW. The latter two conditions may become the basis for the long-term effects of TAI existence in the mothers on the health of offspring. PTB is not only an important cause of perinatal infant death but may also lead to some severe complications (*e.g.*, cerebral palsy, retinopathy, and encephalorrhagia), with a prevalence ranging from 5% to 18% of all births in northern China [32]. The development cannot be well predicted. LBW has been known to have in close relationship with metabolic disorders (*e.g.*, obesity and insulin resistance) during childhood and adolescence [33, 34]. Although previous studies have investigated the associations between TAI and the occurrence of PTB/LBW, there is still a lack of consistent findings [9, 10, 23, 35, 36], and the related mechanisms are unclear. It is assumed that PTB and LBW were caused by an impaired thyroidal response to Human Chorionic Gonadotropin (HCG), alterations in thyroid function, or inflammation. Women with TAI in pregnancy exhibited an impaired thyroidal response to HCG, and an inadequate FT4 response to HCG has been related to an increased risk of PTB and LBW [37, 38]. Oztas *et al*. found that plasma interleukin-6 levels were significantly increased in TAI females and associated with PTB and LBW [39]. In this study, trend tests have further demonstrated the association between serum TPOAb and TgAb expression in T3 and PTB/LBW occurrence in euthyroid women, which has never been reported before. We found that the proportions of PTB and LBW in the offspring of euthyroid females progressively raised with increasing serum TPOAb and TgAb titers. It suggests there are potential interactions between TPOAb and TgAb expressions or some other underlying auto-antibodies indeed involved in the occurrence of PTB and LBW among TAI women, as previously reported on miscarriage [40, 41].

The strengths of this study are listed as follows: It has roughly exhibited the prevalence rates of different TDs throughout the entire pregnancy in the natural pregnant population without any related interventions and is the first study to analyze the dynamic changes of their thyroid parameters across the entire pregnancy. It has simultaneously evaluated the respective contribution of those TDs and anti-thyroid antibody expressions during each of the three trimesters to the development of common adverse obstetric outcomes. There are 4 main studies on the relationship between TDs across the three trimesters and the risk of some common adverse obstetric outcomes. However, none of them has simultaneously evaluated the respective influences of TDs existent in each trimester on the developments of common adverse maternal and perinatal outcomes (Supplementary Table **2**) [12-15].

## CONCLUSION

Taken together, the percentage of euthyroidism exhibited a significant increasing trend while that of thyrotoxicosis gradually decreased from T1 to T3 among non-TAI pregnant women but not in TAI ones from the natural population. Hypothyroxinemia was the most common thyroid disorder among non-TAI women in T1 and T2, whereas the prevalence of SCT was highest among both TAI and non-TAI women in T3. TAI and hypothyroidism in T1 were independently associated with a higher risk of GHT, SCH, and SCT in T1 were independent risk factors for postpartum hemorrhage and SGA, respectively. TPOAb^+^/TgAb^+^ in T3 was an independent risk factor for both PTB and LBW. These findings support the universal screening of thyroid functions and its auto-antibody production in early pregnancy. However, there is no need for excessive concern and treatment, although it is necessary to monitor thyroid functions and its auto-antibody production during the whole pregnancy. A larger multicenter cohort study is awaited to further confirm the findings, and a series of experiments need to be performed to explore the related mechanisms.

## Figures and Tables

**Table 1:** The general characteristics and thyroid parameters of the participants and their adverse pregnancy outcomes.

-	Non-TAI women (n=344)	TAI women (n=46)
**Maternal characteristics**	-	-
Maternal age (years)^a^	29(27-31)	30(26-33)
Gestational age at enrollment (weeks)^a^	12(10-13)	12(10-12)
College and above education, n(%)^b^	164(47.7%)	18(39.1%)
Han nationality, n(%)^b^	296(86.0%)	43(93.5%)
Maternal BMI before pregnancy (kg/m^2^)^a^	21.29(19.56-23.81)	21.63(19.69-23.75)
History of alcohol intake, n(%)^b^	17(4.9%)	6(13.0%)^*^
History of smoking, n(%)^b^	11(3.2%)	2(4.3%)
History of tea consumption, n(%)^b^	19(5.5%)	2(4.3%)
History of coffee consumption, n(%)^b^	36(10.5%)	6(13.0%)
Multipara, n(%)^b^	49(14.2%)	11(23.9%)
Mode of delivery^b^	-	-
Vaginal delivery, n(%)	56(16.3%)	9(19.6%)
Caesarean delivery, n(%)	158(45.9%)	25(54.3%)
Operative vaginal delivery, n(%)	130(37.8%)	12(26.1%)
FBG, mmol/L^^a^^	-	-
T1	4.22(3.96-4.47)	4.13(3.99-4.34)
T2	4.00(3.79-4.23)	3.98(3.78-4.14)
T3	3.96(3.71-4.23)	3.92(3.67-4.05)
FT4, pmol/L^c/d^	-	-
T1	16.24±2.46	15.75±2.18
T2	13.35±1.69^##^	13.90±1.90^##^
T3	11.68±1.52^##/††^	12.11±1.52^##/††^
FT3, pmol/L^c/d^	-	-
T1	4.78±0.69	4.63±0.54
T2	4.28±0.51^##^	4.30±0.64^#^
T3	3.95±0.53^##/††^	3.92±0.56^**/##/††^
TSH, mIU/L^a/e^	-	-
T1	1.29(0.63-1.89)	1.81(1.11-3.04)
T2	1.71(1.24-2.45)^##^	2.11(1.33-2.86)
T3	1.84(1.26-2.43)^##^	2.01(1.11-2.95)
TPOAb, IU/mL^a/e^	-	-
T1	9.12(7.15-11.72)	68.03(17.75-163.45)^**^
T2	9.25(7.60-11.17)	41.52(12.35-117.90)^**^
T3	9.48(7.56-11.40)	23.35(10.34-71.69)^*/#^
TgAb, IU/mL^a/e^	-	-
T1	15.00(13.60-16.74)	200.75(103.46-450.20)^**^
T2	14.85(13.28-16.46)	133.85(37.40-426.58)^**^
T3	14.68(12.94-16.36)^#^	69.09(19.98-233.53)^##^
^GHT^, n(%)^b^	13(3.8%)	5(10.9%)^*^
^GDM^, n(%)^b^	45(13.1%)	7(15.2%)
Oligohydramnios, n(%)^b^	40(11.6%)	3(6.5%)
^PROM^, n(%)^b^	78(22.7%)	9(19.6%)
Postpartum hemorrhage, n(%)^b^	11(3.2%)	2(4.3%)
**Fetal characteristics**	-	-
Term at delivery (weeks)^a^	39(38-40)	39(38-40)
Birth weight (kg)^a^	3.40(3.15-3.70)	3.30(2.95-3.56)
Birth length (cm)^a^	53(52-54)	53(51-53)
Blood glucose readings (mmol/L)^a^	4.10(3.50-4.80)	3.80(3.25-4.75)
PTB, n(%)^b^	12(3.5%)	4(8.7%)
LBW, n(%)^b^	7(2.0%)	4(8.7%)^*^
Macrosomia, n(%)^b^	36(10.5%)	3(6.5%)
Fetal distress, n(%)^b^	30(8.7%)	7(15.2%)
SGA, n(%)^b^	11(3.2%)	0(0%)

**Note:** Normally-distributed continuous variables were expressed as mean±standard deviation, skewed distributed variables were expressed as median (25-75^th ^percentiles), categorical variables were expressed using the number of cases and composition ratio n (%).

Statistical analyses were performed using ^a^Mann-Whitney U test, ^b^chi-squared test or Fisher’s exact test, ^c^independent sample t-test, ^d^analysis of variance, and ^e^Kruskal-Wallis test, respectively. The Bonferroni test was used for *post-hoc* analysis.

As compared with that of non-TAI, ^* ^*P* < 0.05, ^** ^*P* < 0.01; as compared with that of the first trimester in the same group, ^# ^*P* < 0.05, ^##^: *P* < 0.01; as compared with that of the second trimester in the same group, ^† ^*P* < 0.05, ^†† ^*P* < 0.01.

**Abbreviation:** TAI, thyroid autoimmunity; BMI, body mass index; T1, the first trimester of pregnancy; T2, the second trimester of pregnancy; T3, the third trimester of pregnancy; FBG, fasting blood glucose; FT4, free thyroxin; FT3, free triiodothyronine; TSH, thyroid stimulating hormone; TPOAb, thyroid peroxidase antibody; TgAb, thyroglobulin antibody; GHT, gestational hypertension;GDM, gestational diabetes mellitus; PROM, premature rupture of membranes; PTB, preterm birth; LBW, low birth weight; SGA, small for gestational age.

**Table 2:** The prevalence rates of thyroid diseases in the three trimesters of pregnancy.

	Euthyroidism n(%)^a^	Hypothyroxinemia n(%)^a^	Hypothyroidism n(%)^a^	SCH n(%)^a^	Thyrotoxicosis n(%)^a^	SCT n(%)^a^
**Non-TAI women (n=344)**						
T1	303(88.1)	24(7.0)	0(0)	1(0.3)	10(2.9)	6(1.7)
T2	319(92.7)^#^	10(2.9)^#^	0(0)	7(2.0)	0(0)^##^	8(2.4)
T3	323(93.9)^#^	7(2.0)^##^	0(0)	1(0.3)	0(0)^##^	13(3.8)
**TAI women (n=46)**						
T1	38(82.5)	4(8.7)	1(2.2)	1(2.2)	1(2.2)	1(2.2)
T2	42(91.3)	1(2.2)	0(0)	2(4.3)	1(2.2)	0(0)
T3	42(91.3)	1(2.2)	0(0)	0(0)	0(0)	3(6.5)

**Note:** ^a ^Statistical analyses were performed using Chi-square test or Fisher’s exact test.

As compared with that of the first trimester, ^# ^*P* < 0.05, ^## ^*P* < 0.01; as compared with that of the second trimester, ^†^*P* < 0.05.

**Abbreviation:** SCH, subclinical hypothyroidism; SCT, subclinical thyrotoxicosis; the others as described in Table **1**.

**Table 3:** Occurrence rates of adverse obstetric outcomes in women with TDs in each trimester.

-	Euthyroid during T1, T2, and T3 n(%)^a^	T1					T2				T3		
		**Hypothyroxinemia** **n(%)^a^**	**Hypothyroidism** **n(%)^a^**	**SCH** **n(%)^a^**	**Thyrotoxicosis ** **n(%)^a^**	**SCT** **n(%)^a^**	**Hypothyroxinemia** ** n(%)^a^**	**SCH** **n(%)^a^**	**Thyrotoxicosis** **n(%)^a^**	**SCT** **n(%)^a^**	**Hypothyroxinemia** **n(%)^a^**	**SCH** **n(%)^a^**	**SCT** **n(%)^a^**
**Non-TAI women**	**n=283**	**n=24**	**n=0**	**n=1**	**n=10**	**n=6**	**n=10**	**n=7**	**n=0**	**n=8**	**n=7**	**n=1**	**n=13**
GHT	11(3.9%)	2(8.3%)	0(0%)	0(0%)	0(0%)	0(0%)	2(20.0%)	0(0%)	0(0%)	0(0%)	0(0%)	0(0%)	0(0%)
GDM	38(13.5%)	3(12.5%)	0(0%)	0(0%)	1(10.0%)	0(0%)	0(0%)	1(14.3%)	0(0%)	1(12.5%)	1(14.3%)	0(0%)	1(7.7%)
Oligohydramnios	31(11.0%)	6(25.0%)^*^	0(0%)	0(0%)	1(10.0%)	0(0%)	3(30.0%)	1(14.3%)	0(0%)	2(25.0%)	1(14.3%)	0(0%)	2(15.4%)
PROM	67(23.8%)	3(12.5%)	0(0%)	0(0%)	3(30.0%)	2(33.3%)	1(10.0%)	0(0%)	0(0%)	2(25.0%)	3(42.9%)	0(0%)	4(30.8%)
Postpartum hemorrhage	8(2.8%)	1(4.2%)	0(0%)	1(100.0%)^*^	0(0%)	0(0%)	0(0%)	0(0%)	0(0%)	1(12.5%)	0(0%)	0(0%)	0(0%)
PTB	12(4.3%)	0(0%)	0(0%)	0(0%)	0(0%)	0(0%)	0(0%)	0(0%)	0(0%)	0(0%)	0(0%)	0(0%)	0(0%)
LBW	6(2.1%)	0(0%)	0(0%)	0(0%)	2(20.0%)	0(0%)	0(0%)	0(0%)	0(0%)	1(12.5%)	0(0%)	0(0%)	0(0%)
Macrosomia	32(11.3%)	3(12.5%)	0(0%)	0(0%)	1(10.0%)	0(0%)	1(10.0%)	0(0%)	0(0%)	0(0%)	1(14.3%)	0(0%)	0(0%)
Fetal distress	24(8.7%)	2(8.3%)	0(0%)	0(0%)	3(30.0%)	0(0%)	1(10.0%)	1(14.3%)	0(0%)	1(12.5%)	0(0%)	0(0%)	1(7.7%)
SGA	9(3.2%)	0(0%)	0(0%)	0(0%)	0(0%)	1(20.0%)	0(0%)	0(0%)	0(0%)	1(12.5%)	0(0%)	0(0%)	0(0%)
**TAI women**	**n=34**	**n=4**	**n=1**	**n=1**	**n=1**	**n=1**	**n=1**	**n=2**	**n=1**	**n=0**	**n=1**	**n=0**	**n=3**
GHT	2(5.9%)	3(75.0%)^**^	0(0%)	0(0%)	0(0%)	0(0%)	1(100%)	0(0%)	0(0%)	0(0%)	1(100%)	0(0%)	0(0%)
GDM	4(11.8%)	2(50.0%)	0(0%)	0(0%)	0(0%)	0(0%)	0(0%)	0(0%)	1(100.0%)	0(0%)	0(0%)	0(0%)	0(0%)
Oligohydramnios	0(0%)	0(0%)	0(0%)	1(100%)^*^	0(0%)	1(100%)^*^	0(0%)	1(50.0%)	0(0%)	0(0%)	0(0%)	0(0%)	1(33.3%)
PROM	5(14.7%)	2(50.0%)	0(0%)	0(0%)	1(100%)	0(0%)	1(100%)	0(0%)	1(100%)	0(0%)	1(100%)	0(0%)	1(33.3%)
Postpartum hemorrhage	1(2.9%)	1(25.0%)	0(0%)	0(0%)	0(0%)	0(0%)	0(0%)	0(0%)	0(0%)	0(0%)	0(0%)	0(0%)	0(0%)
PTB	3(8.8%)	1(25.0%)	0(0%)	0(0%)	0(0%)	0(0%)	0(0%)	0(0%)	0(0%)	0(0%)	0(0%)	0(0%)	0(0%)
LBW	3(8.8%)	1(25.0%)	0(0%)	0(0%)	0(0%)	0(0%)	0(0%)	0(0%)	0(0%)	0(0%)	0(0%)	0(0%)	0(0%)
Macrosomia	3(8.8%)	0(0%)	0(0%)	0(0%)	0(0%)	0(0%)	0(0%)	0(0%)	0(0%)	0(0%)	0(0%)	0(0%)	0(0%)
Fetal distress	5(14.7%)	1(25.0%)	1(100.0%)	0(0%)	0(0%)	0(0%)	0(0%)	0(0%)	0(0%)	0(0%)	0(0%)	0(0%)	0(0%)
SGA	0(0%)	0(0%)	0(0%)	0(0%)	0(0%)	0(0%)	0(0%)	0(0%)	0(0%)	0(0%)	0(0%)	0(0%)	0(0%)

**Note:** There were no hypothyroidism existent during T2 and T3, no thyrotoxicosis during T3 in all the participants.

^a ^Statistical analyses were performed using Chi-square test or Fisher’s exact test.

As compared with that of euthyroid group during the whole pregnancy, ^*^P < 0.05, ^**^P < 0.01.

The abbreviations as described in Table **1** and Table **2**.

## Data Availability

The data and supportive information are available within the article.

## LIST OF ABBREVIATIONS

TPOAb: Anti-thyroid Peroxidase Antibody
TgAb: Anti-thyroglobulin Antibody
T1: First Trimester
TH: Thyroid Hormone
TDs: Thyroid Dysfunctions
FT3: Free Triiodothyronine
TSH: Thyroid Stimulating Hormone
TAI: Thyroid Autoimmunity
FT4: Free Thyroxine
GHT: Gestational Hypertension
GDM: Gestational Diabetes Mellitus
MFCNC: Maternal-fetal Cohort Study in Northeast China
OGTT: Oral Glucose Tolerance Test
FBG: Fasting Blood Glucose
SCH: Subclinical Hypothyroidism
SCT: Subclinical Thyrotoxicosis
PROM: Premature Rupture of Membranes
LBW: Low Birth Weight
SGA: Small for Gestational Age
