# Supplementary material for: The Dynamic Changes in Maternal Thyroid Parameters Across the Three Trimesters and Their Differential Effects on the Occurrence of Adverse Obstetric Outcomes
Source: Endocr Metab Immune Disord Drug Targets. 2025 Mar 4;26:e18715303351648. doi: 10.2174/0118715303351648250120113846 (PMC13555757; doi:10.2174/0118715303351648250120113846)
Supplement: Supplementary file 1 [file EMIDDT-26-E18715303351648_SD1.pdf]

# Supplementary Material

## The Dynamic Changes in Maternal Thyroid Parameters Across the Three Trimesters and Their Differential Effects on the Occurrence of Adverse Obstetric Outcomes

Zheng Yang[1, 2,†], Jingli Sun[3,†], Jinguang Wang[3], Xiaohui Jin[1], Yiyang Gao[1], Zhenyu Lin[1], Chenling Fan[1], Dongdong Luo[1], Deping Wang[1], Weiping Teng[1], Zhongyan Shan[1,*] and Jing Li[1,*]

[1]*Department of Endocrinology and Metabolism, Institute of Endocrinology, Liaoning Provincial Key Laboratory of Endocrine Diseases, NHC Key Laboratory of Diagnosis and Treatment of Thyroid Diseases, The First Hospital of China Medical University, Shenyang, 110001, P. R. China;* [2]*Department of Endocrinology, Nanyang Central Hospital, Henan 473000, P. R. China;* [3]*Department of Obstetrics and Gynecology, General Hospital of Northern Theater Command (Heping Campus), Shenyang, 110001, P. R. China*

Supplementary Table 1. The trimester-specific reference ranges of serum TSH and FT4 for the pregnant women established by Liu J et al. [20]

| | TSH (mIU/L) | | | FT4 (pmol/L) | | |
|---|---|---|---|---|---|---|
| | 2.5th | median | 97.5th | 2.5th | median | 97.5th |
| T1 | 0.09 | 1.47 | 4.52 | 13.15 | 17.02 | 20.78 |
| T2 | 0.45 | 1.93 | 4.32 | 9.77 | 13.64 | 18.89 |
| T3 | 0.71 | 2.25 | 5.46 | 8.72 | 11.97 | 15.37 |

Abbreviation: P2.5: 2.5th percentile; P97.5: 97.5th percentile, the others as described in Table 1.

Serum FT4, TSH were measured using electrochemiluminescence immunoassays with Cobas Elecsys 601 (Roche Diagnostics, Switzerland)

Supplementary Table 2. Summary of the main studies on the relationship between thyroid disorders in the three trimesters and the risk of common adverse obstetric outcomes

| References | Subjects | Thyroid parameters and gestational age at the time of measurement | Outcomes of concern | Methods | Results |
|---|---|---|---|---|---|
| Monen et al.[12] | 1051 healthy Caucasian women | TSH, FT4, TPOAb at 12, 24, and 36 weeks of pregnancy | SGA | High serum maternal TSH during gestation as an independent variable (>97.5th percentile at one or more trimesters). | TSH ≥ 97.5th percentile at one or more trimesters was related to an increased risk of SGA |
| Nishioka et al. [13] | 163 normal pregnant women | TSH, FT4, FT3 at 12, 25, and 36 weeks of pregnancy | LBW | The multivariate linear regression analysis was performed between birth weight and maternal $\Delta TSH_{12–36W,}$ which was obtained by subtracting $TSH_{12W}$ from $TSH_{36W}$ | $\Delta TSH_{12–36W}$ was inversely correlated with birth weight |
| Kiran et al. [15] | 718 pregnant women | TSH, FT4 in 1st, 2nd and 3rd trimesters | GDM, GHT, postpartum hemorrhage | Univariate analysis of maternal outcomes between the group with TSH > 2.5 mIU/L and that with TSH ⩽ 2.5 mIU/L | Postpartum hemorrhage was significantly associated with serum TSH > 2.5 mIU/L in the preconception and third trimester |
| Johns et al.[14] | 439 pregnant women | TSH, FT4, TT3, TT4 at visit 1 (range: 4.7 to 19.1 weeks of pregnancy), visit 2 (range: 14.9 to 32.1 weeks), visit 3 (range: 22.9 to 36.3 weeks), and visit 4 (range: 33.1 to 38.3 weeks) | PTB | Logistic regression was used to explore the associations between increases in individual thyroid hormones (In-TSH, In-FT4, TT4, TT3 in each trimester, respectively) and the odds of PTB | PTB was positively associated with serum TT3 at visit 1 and visit 3, but negatively associated with serum FT4 at visit 3. |
| The current study | 390 pregnant women | TSH, FT4, TPOAb, TgAb in T1, T2, and T3 | GHT, GDM, oligohydramnios, PROM, postpartum hemorrhage, PTB, LBW, macrosomia, fetal distress, SGA | 1)Thyroid disorders during each of the three trimesters were simultaneously included into binary logistic regression analysis with stepwise procedure<br>2)Serum $TPOAb^+/TgAb^+$ expressions during each of the three trimesters were simultaneously included into binary logistic regression analysis with stepwise procedure among only euthyroid women | 1)TAI and hypothyroxinemia in T1 was independently associated with an increased risk of GHT<br>2)SCH in T1 was an independent risk factor for postpartum hemorrhage<br>3)SCT in T1 was an independent risk factor for SGA<br>4)$TPOAb^+/TgAb^+$ in T3 was an independent risk factor for both PTB and LBW. |

The abbreviations as described in Table 1 and Table 2.
